# Une cause rare et atypique des ulcérations scrotales

**DOI:** 10.11604/pamj.2018.29.87.14511

**Published:** 2018-01-30

**Authors:** Mohamed El Amraoui, Naoufal Hjira

**Affiliations:** 1 Service de Dermatologie-Vénéréologie, Hôpital Militaire d’Instruction Mohammed V, Rabat, Maroc

**Keywords:** Ulcérations, scrotales, fièvre typhoïde, Ulcerations, scrotal, typhoid fever

## Abstract

Salmonelloses are bacterial infections of the faecal peril; they are endemic in some regions of the world. They can colonize almost any organ hematogenously, hence, severe forms may be observed. We report a case of typhoid fever in a 16-year old young adolescent with no medical history, sexually transmitted diseases or sexual intercourses, presenting with necrotic scrotal ulcerations evolving over the past 3 weeks in a context of fever, diarrhoea, weight loss and alteration of the general state. Paraclinical assessment showed 7g/dl hypochromic microcyticanemia with target cells in blood smear and normal hemoglobin electrophoresis, biologic inflammatory syndrome, malabsorption syndrome, cytolysis and hepatic cholestasis 20 times above the normal, hepatosplenomegaly, normal viral serologic testing (HBV, HCV, HIV, EBV and CMV) and positive testing for salmonella. The patient underwent 3 weeks of therapy with fluoroquinolones with favorable evolution. Dermatologic manifestations are frequent and polymorphic in typhoid fever; however cutaneous and, in particular, scrotal ulcerations are rarely reported. Differential diagnosis may be based on sexually transmitted diseases, inflammatory bowel diseases or hematologic disorders. More often diarrhoea is reduced or absent and patient’s outcome depends on antibiotic. Diagnostic confirmation in patients with an atypical clinical picture, as in the case of our patient, is based on serology. Treatment is based on proper and prolonged antibiotic therapy and on prophylaxis measures to neighbouring.

## Image en médecine

Les salmonelloses sont des infections bactériennes du péril fécal, endémiques dans certaines régions du monde. Elles peuvent coloniser pratiquement tous les organes par voie hématogène, ainsi, des formes sévères peuvent être observées. Nous rapportons le cas de multiples ulcérations scrotales, nécrotiques, chez un jeune adolescent ayant révélé une fièvre typhoïde. Jeune adolescent âgé de 16 ans, sans antécédents pathologiques notables, notamment sans notion d'IST ou de rapports sexuels. A consulté pour des ulcérations scrotales, nécrotiques, évoluant depuis 3 semaines dans un contexte de fièvre, de diarrhée, d'amaigrissement et d'altération de l'état général. Le bilan para clinique montrait une anémie microcytaire hypochrome à 7g/dl avec des globules rouges en cible au frottis et une électrophorèse de l'hémoglobine normale, un syndrome inflammatoire biologique, un syndrome de malabsorption, une cytolyse et une choléstase hépatique à 20 fois la normale, une hépato-splénomégalie, les sérologies virales (VHB, VHC, HIV, EBV et CMV) étaient normales, alors que les sérologies des salmonelles étaient positives. Le patient a été mis sous Fluor quinolones pendant 3 semaines avec une évolution favorable. Les manifestations dermatologiques de la fièvre typhoïde sont fréquentes et polymorphes, cependant les ulcérations cutanées et particulièrement scrotales sont rarement rapportées. Le diagnostic différenciel peut se faire avec les IST les MICI ou les hémopathies. Le plus souvent la diarrhée est fruste ou absente et mise au dépend de l'antibiothérapie. La confirmation dans les tableaux atypiques, comme chez notre patient, fait appel aux sérologies. Le traitement repose sur une antibiothérapie adaptée et prolongée et une prophylaxie pour l'entourage.

**Figure 1:**
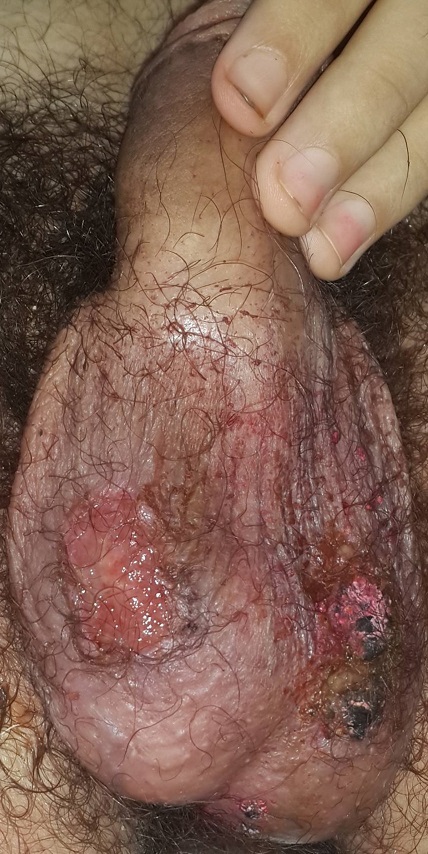
Ulcérations nécrotiques scrotales révélant une fièvre typhoïde

